# HPV vaccine-related thyroid adverse events: temporal patterns and reporting trends

**DOI:** 10.3389/fphar.2025.1664697

**Published:** 2025-11-11

**Authors:** Liying Song, Jingkai Di, Jiaolin Fan, Rongrong Xi, Siyu Liang, Guosheng Duan, Yuanzheng Ding, Shuai Hao, Jing Liu

**Affiliations:** 1 Department of Thyroid Surgery, First Hospital of Shanxi Medical University, Taiyuan, China; 2 Department of Orthopedics, The Second Hospital of Shanxi Medical University, Taiyuan, China; 3 School of Basic Medical Sciences, Shanxi Medical University, Taiyuan, China; 4 Second Clinical Medical College, Shanxi Medical University, Taiyuan, China; 5 Department of Radiotherapy of the Fifth Clinical Medical College of Shanxi Medical University, People’s Hospital of Shanxi Province, Taiyuan, Shanxi, China

**Keywords:** HPV vaccine, thyroid adverse events, pharmacovigilance, VAERS database, thyroid

## Abstract

**Background:**

Human papillomavirus (HPV) vaccination is essential for cervical cancer prevention, but concerns about thyroid-related adverse events (AEs) have emerged.

**Methods:**

This pharmacovigilance study aimed to assess potential associations between HPV vaccination and thyroid disorders using spontaneous report data. Reports from the U.S. Vaccine Adverse Event Reporting System through 31 December 2024, were analyzed. Disproportionality analyses (PRR, ROR, BCPNN, MGPS) were performed, with subgroup analyses by gender, age, and vaccine type. A Weibull shape parameter model assessed the temporal risk pattern.

**Results:**

Among 60,840 HPV vaccine-related reports, 13 thyroid-associated AEs showed positive signals. Hypothyroidism (ROR = 11.65) and autoimmune thyroiditis (ROR = 4.26) were the strongest signals. Most cases occurred in females under 65 years. HPV-4 was linked to 72.8% of thyroid AEs. The cumulative reporting rate reached 69.1% within 180 days.

**Conclusion:**

Our findings suggest a potential association between HPV vaccination and thyroid disorders, notably hypothyroidism and autoimmune thyroiditis. Continued pharmacovigilance and further mechanistic investigations are needed.

## 1 Introduction

Human papillomavirus (HPV) is a double-stranded DNA virus that infects epithelial cells and evades immune surveillance by modulating host immune responses, thereby promoting oncogenesis (Steinbach and Riemer, 2018; Yousefi et al., 2021). Clinical manifestations encompass benign lesions caused by low-risk carcinogenic HPV subtypes (e.g., genital, anal, oral, or laryngeal warts) and malignancies associated with high-risk carcinogenic subtypes (e.g., vulvovaginal, oropharyngeal, cervical, and head/neck cancers) (Sharma et al., 2023; Yousaf et al., 2024).

HPV vaccination is currently one of the most effective preventive measures against cervical cancer. According to World Health Organization (WHO) data, 133 countries/regions have incorporated HPV vaccines into their National Immunization Programs (NIPs) (Yousaf et al., 2024). Three prophylactic HPV vaccines are commercially available (2vHPV, 4vHPV, 9vHPV), all containing the major viral capsid protein L1 derived from specific HPV subtypes. These proteins are produced via recombinant DNA technology and self-assemble into virus-like particles (VLPs). VLPs lack viral DNA and live infectious particles, rendering them non-infectious and non-carcinogenic. The vaccines utilize L1 protein as target antigens, inducing humoral immunity to generate virus-neutralizing antibodies with 10- to 100-fold higher titers than natural infection, thereby effectively neutralizing virions (Yousefi et al., 2021).

Although the vaccines are generally safe, rare adverse events have been reported, including autoimmune conditions such as premature ovarian insufficiency (POI), Guillain–Barré syndrome, and complex regional pain syndrome (Tsai et al., 2023). Emerging studies have suggested potential associations between HPV vaccination and thyroid disorders (Willame et al., 2020). However, findings are inconsistent, with some studies suggesting increased risk, while others report null or even protective associations (Grimaldi-Bensouda et al., 2017; Hviid et al., 2018; Rosillon et al., 2020; Yoon et al., 2021).

The Vaccine Adverse Event Reporting System (VAERS), a national spontaneous surveillance system, accepts reports of potential vaccine-related adverse events (AEs) from healthcare professionals, vaccine recipients, manufacturers, and other reporters (Vaccine Adverse Events Reporting System. VAERS data use guide). Trained medical coders systematically review all reports, classifying signs and symptoms using Medical Dictionary for Regulatory Activities (MedDRA), a clinically validated international standardized terminology (Shimabukuro et al., 2015). Based on this, data mining analysis of adverse reactions using the VAERS database may provide a preliminary validation strategy for the study of the relationship between HPV vaccines and AEs.

This study aimed to systematically analyze thyroid-related adverse events reported after HPV vaccination in VAERS, focusing on their frequency and temporal patterns, to provide evidence supporting future research and public health risk assessment.

## 2 Materials and methods

VAERS was established in 1990 as a national spontaneous reporting system for post-vaccination adverse events. It is jointly administered by the Centers for Disease Control and Prevention (CDC) and the Food and Drug Administration (FDA) in the United States (Sever et al., 2004; Moro et al., 2023). Reports are submitted to VAERS by patients, clinicians, regulators, and others. It is imperative that submissions accurately reflect the information regarding the administered vaccine, the individual who received the vaccine, and the adverse events encountered by the vaccinated person (Chen et al., 2024). Reports are categorised as either serious or non-serious. Incidents of death, life-threatening illness, hospitalisation, or prolongation of existing hospitalisation, or permanent disability must be classified as serious, as defined by the Code of Federal Regulations (Myers et al., 2020). The standardisation of symptoms reported in VAERS is carried out by third-party experts according to the standard International Medical Dictionary of Terms (MedDRA) (Zhou et al., 2003), a dictionary of medical terms used for the reporting and monitoring of adverse drug reactions. The dictionary is organised into five levels, from broad to specific, and includes Lowest Level Terminology (LLT), Preferred Terminology (PT), Higher Level Terminology (HLT), Higher Level Group Terminology (HLGT), and 27 System Organ Class (SOC). This classification and standardisation have been shown to improve the efficiency of data analysis and signal detection (Bousquet et al., 2005). In this study, an initial set of thyroid-related Preferred Terms (PTs) was derived from relevant narrow scope Structured MedDRA Queries (SMQs). This set was subsequently refined by two independent clinical endocrinologists to ensure clinical relevance and alignment with the study objective.

The search was conducted on the database provided by VAERS for data with an initial cut-off date of 31 December 2024. The raw data files, including VAERSDATA, VAERSVAX, and VAERSSYMPTOMS, were downloaded in CSV format and subsequently collated and merged. The report was subject to screening in accordance with two conditions: firstly, the presence of HPV vaccination (HPV2, HPV4, HPV9, HPVX). Secondly, the presence of thyroid-related AE was taken into consideration. The selection statistics collated baseline information (state/region, age, gender, time from vaccination to adverse event, serious vs. non-serious, etc.), vaccine information (type, dose, number of times vaccinated, etc.), clinical outcomes, and annual uptake frequency statistics; and time to induction was statistically grouped.

Multiple pharmacovigilance signal detection algorithms were applied in order to validate potential positive signals for thyroid-associated adverse events following HPV vaccination. These included the proportional reporting ratio (PRR), the reporting reporting odds ratio (ROR), the Bayesian confidence propagation neural network (BCPNN), and the Multi-item Gamma Poisson Shrinker (MGPS) algorithm. The underlying principles of these methodologies are predicated upon a 2x2 columnar table (wherein ‘a' denotes target ADE occurrence, ‘b' denotes target ADE non-occurrence, ‘c' denotes target vaccination, and ‘d' denotes non-vaccination or vaccination with other vaccines). Supplementary Table S1 provides detailed formulas for this analytical method and the thresholds for positive signals. According to Toshiyuki Sakaeda et al., when at least one of the four indicators met the aforementioned criteria, the adverse event was classified as drug-related (Sakaeda et al., 2013) and could be further reviewed. All analyses were performed using R (version 4.4.3) in RStudio.

## 3 Results

### 3.1 General report characteristics

**FIGURE 1:**
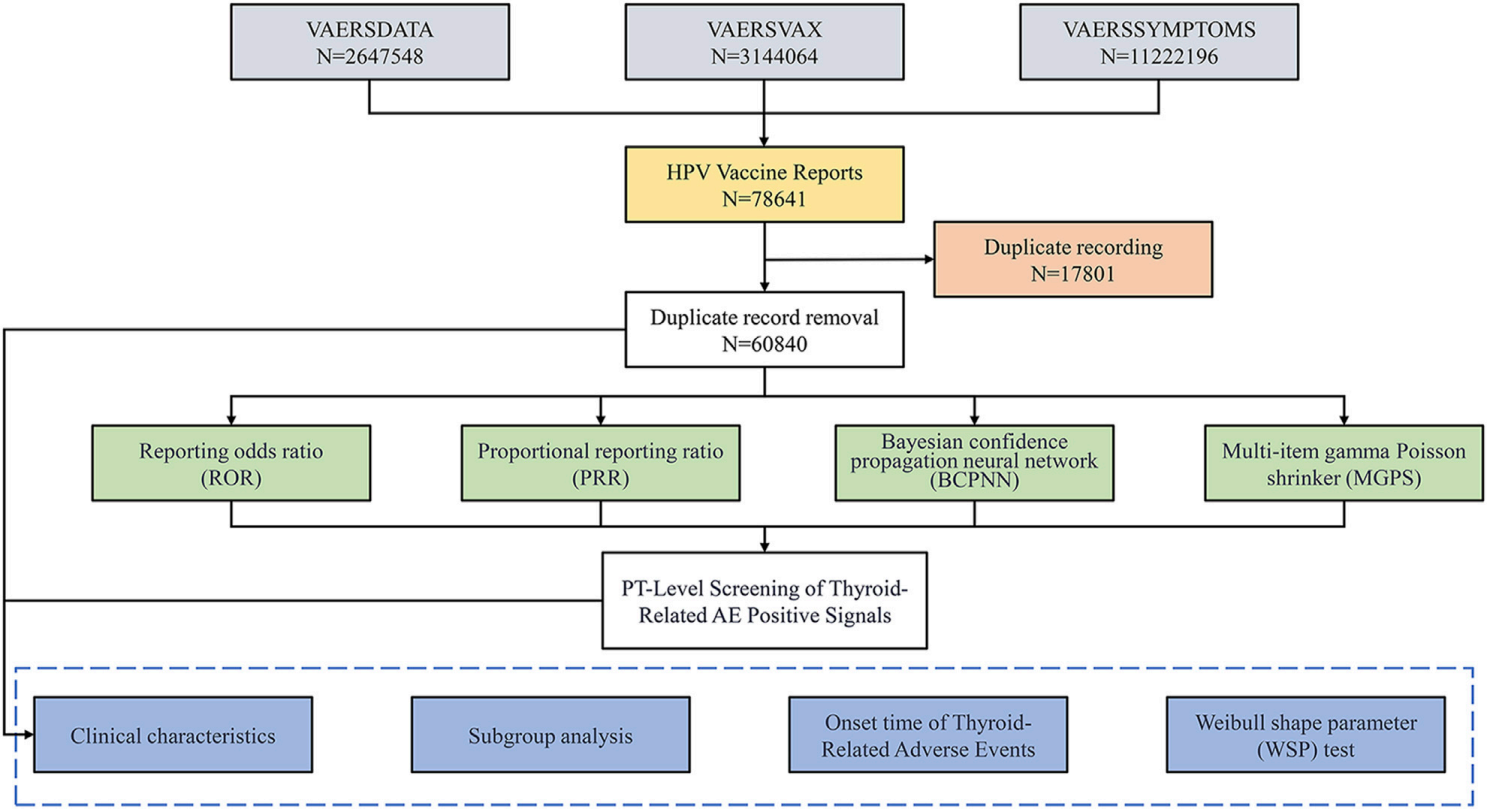
Flowchart for Selecting HPV Vaccine-Related Adverse Events from the VAERS Database. This flowchart outlines the process for identifying adverse events (AEs) related to the HPV vaccine using data from the Vaccine Adverse Event Reporting System (VAERS). It illustrates the total number of reports, the removal of duplicate records, and the methodologies employed for signal detection, including Reporting Odds Ratio (ROR), Proportional Reporting Ratio (PRR), Bayesian Confidence Propagation Neural Network (BCPNN), and Multi-item Gamma Poisson Shrinker (MGPS). Finally, it details the PT-level screening focused on identifying thyroid-related AEs through various analytical approaches.

Figure 1 illustrates the data processing and analysis flow. As of 31 December 2024, the VAERS database contained 2,647,548 case reports and 11,222,196 individual adverse events. Among these, 78,641 reports were identified as HPV vaccine-related. After deduplication, 60,840 unique cases involving individuals who received only HPV vaccines were retained for analysis. The distribution of vaccine types was as follows: HPV2 (7.7%), HPV4 (61.3%), HPV9 (27.3%), and unspecified type (HPVX, 3.7%).

Demographic analysis revealed a predominance of female recipients (69.4%), with the most frequent age group being under 18 years (40.5%), followed by those aged 18–65 years (22.8%). A total of 10,349 reports (17.0%) were classified as serious, 65.1% of which were associated with HPV4, and 90.6% occurred in females. Among all HPV-related reports, 579 deaths were recorded, accounting for 1.0% of the total (Table 1). Furthermore, there were 21,844 adverse event reports indicating that they occurred within 30 days after vaccination, which suggests a temporal clustering phenomenon.

**TABLE 1:** Clinical characteristics of reports with HPV vaccine from the VAERS database.

Characteristics	HPV vaccine (N,%)
Total number of reports	60,840
Gender	
Female	42,239 (69.4%)
Male	5703 (9.4%)
Unknown	12,898 (21.2%)
Age (year)	
<18	24,615 (40.5%)
≥18,< 65	13,844 (22.8%)
≥65, <85	59 (0.1%)
≥85	3 (0.0%)
Unknown	22,319 (36.7%)
Clinical Outcome	
Death (DE)	579 (1.0%)
Life-Threatening	970 (1.6%)
Hospitalization	6893 (11.3%)
Prolonged Hospitalization	247 (0.4%)
Disability	3457 (5.7%)
Recovered	
RECOVD-N	14,236 (23.4%)
RECOVD-Y	1248 (2.1%)
RECOVD-U	28,788 (47.3%)
RECOVD-NotAvailable	16,568 (27.2%)

Abbreviation: N, number of adverse event reported.

### 3.2 Signal detection of thyroid-related adverse events

To identify potential associations between HPV vaccination and thyroid-related AEs, four validated signal detection algorithms were applied: ROR, PRR, BCPNN, and MGPS.

**FIGURE 2:**
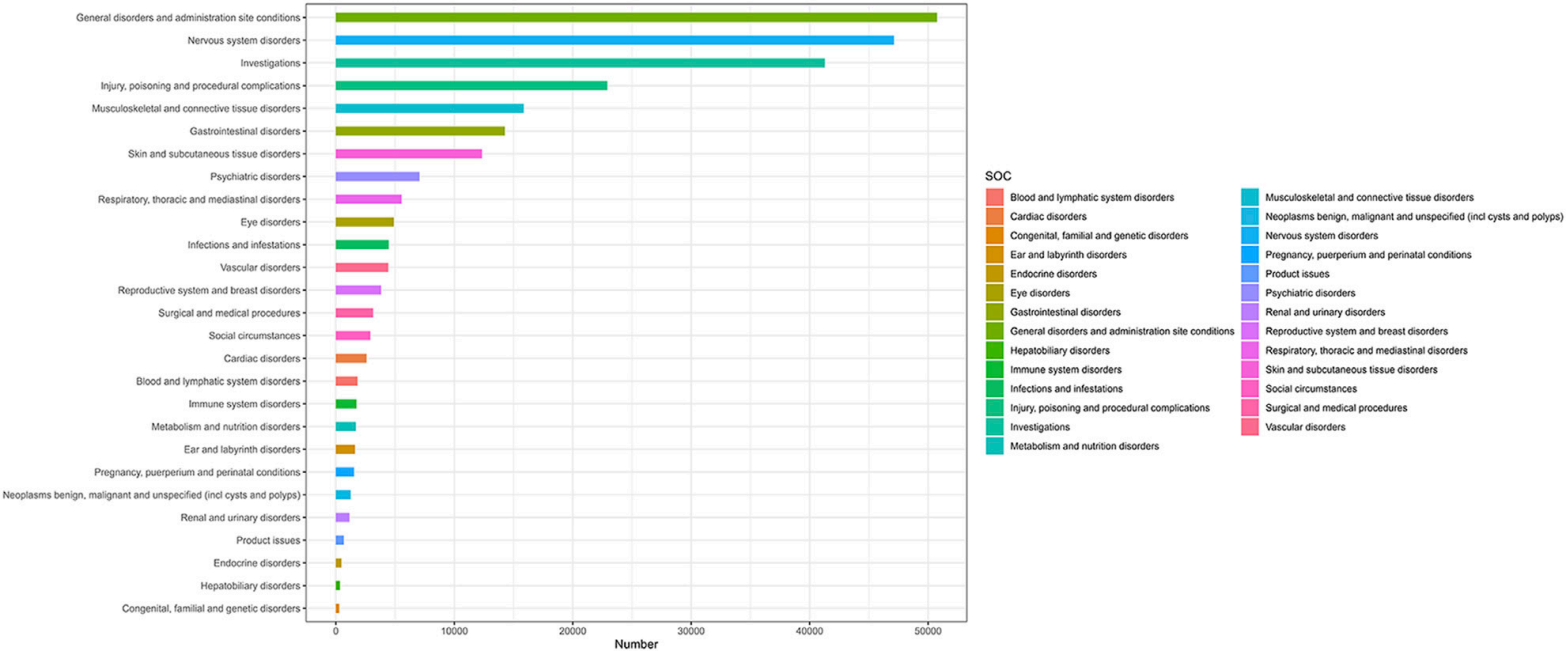
Adverse Event Reports by SOC Following HPV Vaccination. The Fig classifies statistics of adverse events (aes) after HPV vaccination according to SOC. The ordinate lists SOC categories (e.g., neurological disorders, gastrointestinal disorders) and corresponding AE terms. The values on the abscissa represent the number of cases.

Analysis was conducted across 27 SOCs, as defined by MedDRA terminology (Figure 2). Among these, 15 SOCs demonstrated statistically significant positive signals. Notably, four SOCs were identified as having strong signals—detected by at least three of the four algorithms—namely: (1) Pregnancy, puerperium and perinatal conditions; (2) Benign, malignant and unspecified neoplasms (including cysts and polyps); (3) Social circumstances; and (4) Endocrine system disorders.

**FIGURE 3:**
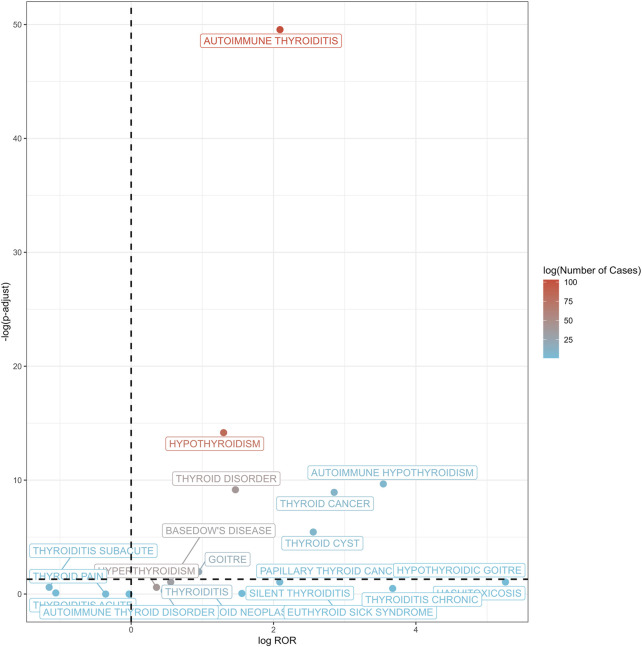
Volcano plot of thyroid-related adverse events post-HPV vaccination. This volcano plot illustrates the association between thyroid-related adverse events and HPV vaccination, with the log of ROR plotted against the negative log of adjusted p-values. Each point represents a specific thyroid condition, with color gradients from blue to red indicating the logarithm of the number of cases reported (log (Number of Cases)). A dashed vertical line denotes an ROR of 1, while a horizontal dashed line indicates a false discovery rate (FDR) adjusted p-value threshold of 0.05.

**FIGURE 4:**
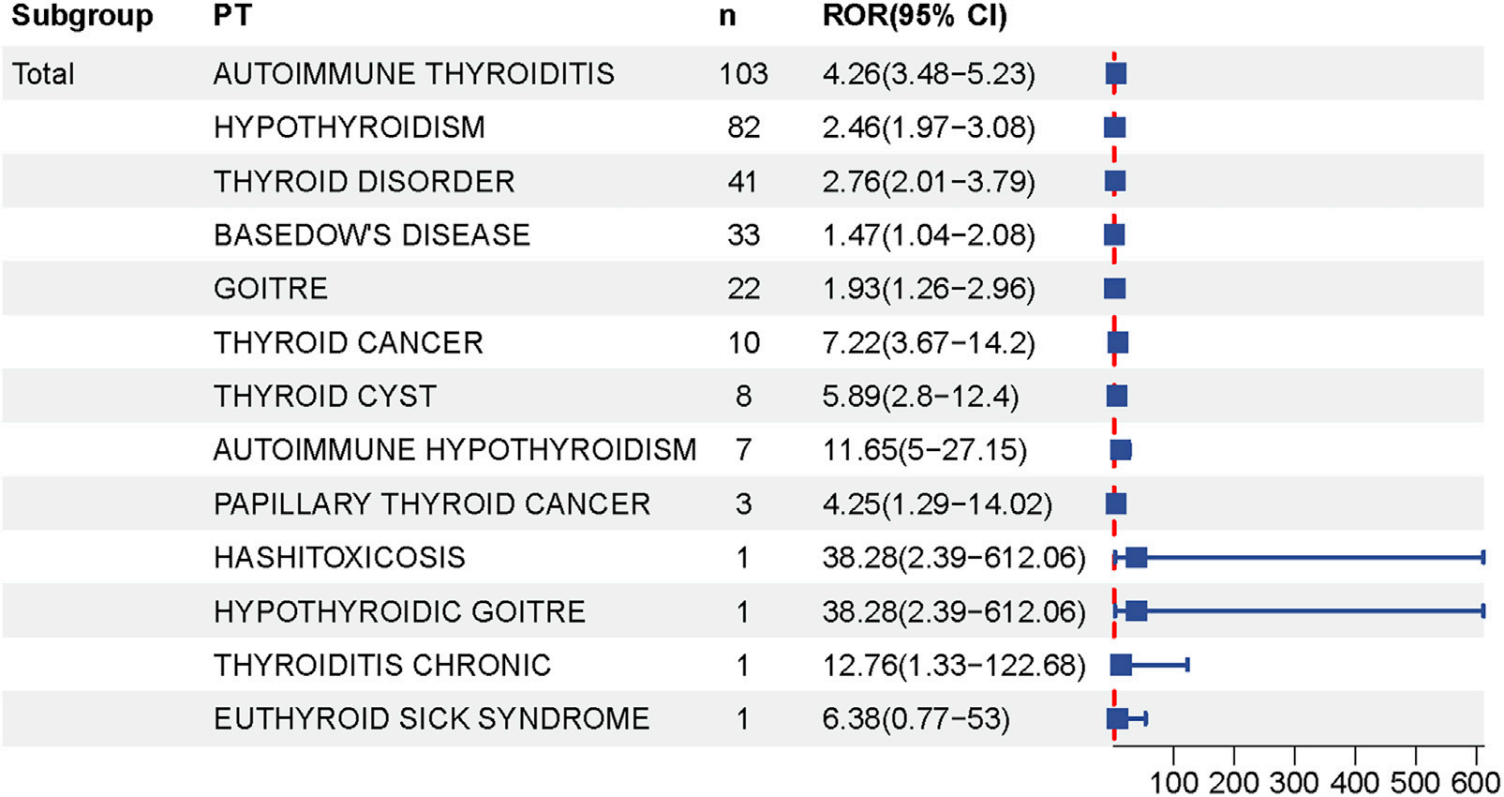
Forest Plot of Thyroid-Related Adverse Events Following HPV Vaccination. This forest plot shows the relative odds ratios (RORs) and 95% confidence intervals (CIs) for various thyroid-related adverse events reported after HPV vaccination. Each square represents the ROR for a particular condition, and the horizontal black line represents the corresponding 95% ci. The dashed line at ROR 1 represents the null hypothesis, indicating no association. Data suggest that risk estimates for some thyroid conditions increase after vaccination.

At the PT level, 13 thyroid-related AEs exhibited statistically significant positive signals (Supplementary Table S2; Figures 3, 4). The most prominent signals were observed for autoimmune thyroiditis (ROR = 4.26, 95% CI: 3.48–5.23; EBGM05 = 3.32) and autoimmune hypothyroidism (ROR = 11.65, 95% CI: 5.00–27.15; EBGM05 = 4.52), with 103 and 82 reported cases, respectively. Additional positive signals included: thyroid cyst (ROR = 5.89), general thyroid disease (ROR = 2.76), hypothyroidism (ROR = 2.46), goiter (ROR = 1.93), Graves’ disease (ROR = 1.47), Hashimoto’s thyrotoxicosis (ROR = 38.28), hypothyroid goiter (ROR = 38.28), chronic thyroiditis (ROR = 12.76), and euthyroid sick syndrome (ROR = 6.38). Importantly, significant signals were also detected for thyroid malignancies, including thyroid cancer (ROR = 7.22) and papillary thyroid carcinoma (ROR = 4.25). Among these, autoimmune thyroiditis, autoimmune hypothyroidism, and thyroid cancer are categorized as important medical events (IMEs).

Subgroup analyses by sex and age (Tables 2, 3) revealed distinct patterns. The vast majority of thyroid-related AEs were reported in female recipients, including autoimmune thyroiditis, autoimmune hypothyroidism, chronic thyroiditis, Hashimoto’s thyrotoxicosis, thyroid cysts, goiter, thyroid cancer, and papillary thyroid carcinoma. Autoimmune thyroiditis was the most frequently reported AE (n = 100). Among male recipients, positive signals were limited to autoimmune hypothyroidism.

**TABLE 2:** Gender-stratified detection of thyroid adverse event signals linked to HPV vaccination.

Subgroup	Preferred terms	N	ROR (95% two-sided CI)	PRR (95% two-sided CI)	χ2	IC (IC025)	EBGM (EBGM05)
Male	AUTOIMMUNE HYPOTHYROIDISM	1	19.41 (2.43–155.24)	19.41 (17.33-21.49)	15.52	4.12 (1.88)	17.37 (3.05)
Female	HASHITOXICOSIS	1	30.25 (1.89–483.71)	30.25 (27.48-33.03)	14.14	3.97 (1.33)	15.63 (1.54)
	THYROIDITIS CHRONIC	1	15.13 (1.37–166.83)	15.13 (12.73-17.53)	8.8	3.38 (0.88)	10.42 (1.4)
	AUTOIMMUNE HYPOTHYROIDISM	6	12.1 (4.7–31.19)	12.1 (11.15-13.05)	43.65	3.16 (1.91)	8.93 (4.04)
	EUTHYROID SICK SYNDROME	1	7.56 (0.85–67.67)	7.56 (5.37-9.75)	4.56	2.64 (0.29)	6.25 (1)
	THYROID CYST	8	5.5 (2.59–11.68)	5.5 (4.75-6.25)	24.93	2.27 (1.23)	4.81 (2.56)
	AUTOIMMUNE THYROIDITIS	100	3.96 (3.22–4.88)	3.96 (3.75-4.17)	195.7	1.85 (1.55)	3.62 (3.04)
	THYROID DISORDER	39	2.62 (1.89–3.64)	2.62 (2.29-2.95)	36.01	1.32 (0.84)	2.49 (1.9)
	HYPOTHYROIDISM	73	2.27 (1.79–2.88)	2.27 (2.03-2.51)	48.37	1.13 (0.78)	2.18 (1.79)
	GOITRE	20	1.85 (1.18–2.91)	1.85 (1.4-2.3)	7.37	0.85 (0.2)	1.8 (1.23)
	THYROID CANCER	10	8.64 (4.28–17.46)	8.64 (7.94-9.35)	52.58	2.8 (1.83)	6.95 (3.86)
	PAPILLARY THYROID CANCER	3	4.78 (1.41–16.14)	4.78 (3.56-5.99)	7.74	2.09 (0.53)	4.26 (1.54)

Abbreviations: CI, confidence interval; EBGM, empirical Bayesian geometric mean; IC, information component; IC025 and EBGM05, lower one-sided for IC, and EBGM, respectively; N, number of adverse event reported; PRR, proportional reporting ratio; ROR, reporting odds ratio; χ2, chi-squared.

^a^ Adhering to the four algorithms.

**TABLE 3:** Age-stratified detection of thyroid adverse event signals linked to HPV vaccination.

Subgroup	Preferred terms	N	ROR (95% two-sided CI)	PRR (95% two-sided CI)	χ2	IC (IC025)	EBGM (EBGM05)
Younger	AUTOIMMUNE HYPOTHYROIDISM	5	127.83 (14.93–1094.21)	127.83 (125.68-129.97)	104.86	4.47 (2.86)	22.14 (3.67)
	EUTHYROID SICK SYNDROME	1	8.52 (0.89–81.93)	8.52 (6.26-10.79)	4.98	2.73 (0.32)	6.64 (1)
	BASEDOW'S DISEASE	24	1.8 (1.19–2.72)	1.8 (1.39-2.21)	7.96	0.8 (0.21)	1.75 (1.24)
	THYROID CYST	8	8.18 (3.69–18.14)	8.18 (7.38-8.98)	38.2	2.69 (1.61)	6.44 (3.31)
	AUTOIMMUNE THYROIDITIS	74	4.66 (3.64–5.97)	4.66 (4.41-4.91)	179.93	2.03 (1.68)	4.1 (3.33)
	THYROID NEOPLASM	2	3.93 (0.89–17.43)	3.93 (2.44-5.42)	3.79	1.82 (0.01)	3.54 (1.02)
	HYPOTHYROIDISM	66	3.38 (2.61–4.36)	3.37 (3.12-3.63)	97.44	1.63 (1.26)	3.1 (2.5)
	THYROID DISORDER	27	2.7 (1.81–4.01)	2.7 (2.3-3.09)	26.07	1.34 (0.77)	2.53 (1.82)
	GOITRE	20	2.06 (1.31–3.25)	2.06 (1.61-2.52)	10.12	0.99 (0.33)	1.98 (1.35)
	THYROID MASS	10	1.95 (1.03–3.71)	1.95 (1.31-2.59)	4.31	0.91 (0.01)	1.88 (1.1)
	HYPERTHYROIDISM	29	1.53 (1.05–2.22)	1.53 (1.15-1.9)	4.95	0.58 (0.04)	1.5 (1.09)
Older	-	-	-	-	-	-	-

Abbreviations: CI, confidence interval; EBGM, empirical Bayesian geometric mean; IC, information component; IC025 and EBGM05, lower one-sided for IC, and EBGM, respectively; N, number of adverse event reported; PRR, proportional reporting ratio; ROR, reporting odds ratio; χ2, chi-squared.

^a^ Adhering to the four algorithms.

No thyroid-related AEs were reported in individuals over the age of 65. All relevant reports originated from patients aged 65 years or younger, with a notable concentration among adolescents under 18 years of age. In this group, commonly reported thyroid-related AEs included autoimmune hypothyroidism (n = 66), thyroid cysts, autoimmune thyroiditis, hypothyroidism, goiter, thyroid tumors, and hyperthyroidism. Autoimmune thyroiditis remained the most frequently reported AE (n = 74). Several previously unreported AEs, such as goiter, hyperthyroidism (n = 29), and thyroid tumors, were also observed. These findings suggest a potential association between HPV vaccination and thyroid-related AEs, particularly in women and younger populations.

In addition, subgroup analysis, stratified by HPV vaccine types, revealed that HPV-4 was associated with the highest absolute number of thyroid-related adverse event (AE) reports (n = 187, 73.0% of total thyroid-related AEs), followed by HPV-9 (n = 54, 21.1%) and HPV-2 (n = 15, 5.9%) (Supplementary Table S3). Autoimmune thyroiditis was the predominant AE subtype across all vaccine groups, accounting for 66.7% (10/15) of AEs in HPV-2 recipients, 35.8% (67/187) in HPV-4 recipients, and 40.7% (22/54) in HPV-9 recipients. Hypothyroidism exhibited a higher reporting proportion in the HPV-4 cohort (32.6%, 61/187), with no comparable signal observed in the HPV-2 or HPV-9 subgroups.

### 3.3 Time-to-onset (TTO) analysis

**FIGURE 5:**
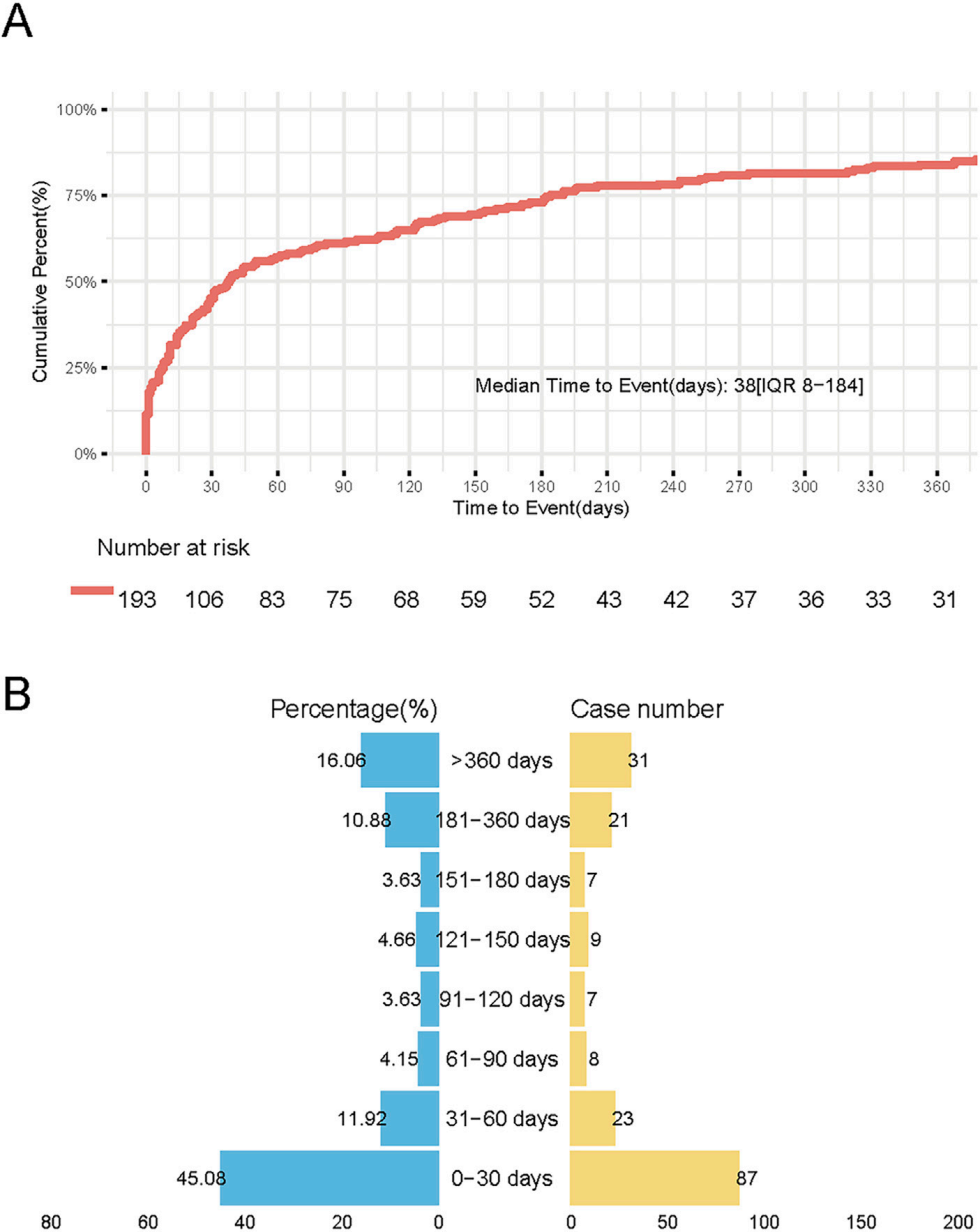
Temporal distribution of Thyroid Adverse Events Following HPV Vaccination. **(A)** Survival curve demonstrating cumulative incidence of thyroid AEs during 360-day follow-up. Vertical axis: Cumulative incidence (%), horizontal axis: Days post-vaccination. **(B)** Butterfly plot showing cumulative incidence of thyroid adverse events (AEs) stratified by post-vaccination intervals. Left panel (blue): Cumulative incidence (%) across sequential time windows Right panel (orange): Absolute case counts per interval.

Reports of thyroid-related AEs following HPV vaccination were primarily concentrated within the first 30 days post-vaccination, accounting for 45.08% of cases. Although the frequency declined over time, a notable proportion (16.06%) of reports still occurred beyond 360 days (Figure 5). This pattern reflects a bimodal distribution, characterized by a peak in early-onset reporting and a secondary rise in the long-term post-vaccination period.

After excluding reports with missing, inaccurate, or unknown onset dates, 68 cases of autoimmune thyroiditis and 4 cases of thyroid cancer were included in the TTO analysis. For autoimmune thyroiditis, the median time to onset was 44 days (interquartile range: 12–248 days). The cumulative reporting rate reached 35.3% within 30 days, increased to 69.1% within 180 days, and remained at 20.6% 1 year after vaccination. Due to the small number of thyroid cancer cases (n = 4), statistical comparison was not performed; the median onset time for these cases was 185.5 days.

The Weibull shape parameter (β) for thyroid-related AEs, calculated using the WSP model, was 0.54 (95% CI: 0.485–0.596) (Table 4), indicating that the reporting risk was highest during the early post-vaccination period and declined thereafter.

**TABLE 4:** Time to onset of HPV vaccine-associated adverse thyroid reaction signals was analysed using the Weibull distribution test.

Time-to-onset (days)			Weibull distribution				Failure type
			Scale parameter		Shape parameter		
N	Median (IQR)	Min–Max	α	95% CI	β	95% CI	Failure type
193	39 (147)	0–4661	151.73	110.62–192.85	0.54	0.49–0.60	Early failure

Abbreviations: CI, confidence interval; IQR, interquartile range; N, number of adverse event reported.

## 4 Discussion

Although HPV vaccination plays a critical role in cervical cancer prevention, concerns remain regarding its potential association with thyroid disorders. This study systematically analyzed thyroid-related AEs following HPV vaccination using the VAERS database. A total of 13 thyroid-related AEs demonstrated statistically significant positive signals, with autoimmune thyroid disease (AITD) and hypothyroidism being the most frequently reported.

The potential association between HPV vaccination and AITD remains a subject of ongoing debate. In our study, positive signals for AITD onset were observed in the context of HPV vaccination. This finding echoes a large cohort study that showed HPV vaccination was statistically significantly associated with an increased risk of autoimmune diseases such as Hashimoto’s thyroiditis (HT) (Hviid et al., 2018). A meta-analysis further supported this association, indicating a 1.5-fold increased risk of AITD after vaccination (Rosillon et al., 2020). However, other studies have reported no significant elevation in thyroiditis risk within 2 years post-vaccination (Castillo-Cano et al., 2022), and a Korean cohort study found no increased risk of HT in either cohort or self-controlled case series analyses (adjusted rate ratio = 1.24; 95% CI: 0.78–1.90; adjusted relative risk = 1.28; 95% CI: 0.53–3.08) (Yoon et al., 2021). Interestingly, Grimaldi-Bensouda et al. observed a decreased risk of AITD in vaccinated girls compared to unvaccinated individuals over a 24-month period (OR = 0.35; 95% CI: 0.13–0.92) (Grimaldi-Bensouda et al., 2017). Our study highlighted a notable signal in the safety data: reports of hypothyroidism and autoimmune thyroiditis following HPV vaccination that we believe merit closer scrutiny and further investigation. Notably, three preferred terms—autoimmune thyroiditis, autoimmune hypothyroidism, and thyroid carcinoma—were classified as IMEs. These findings suggest that individualized vaccination strategies, such as avoiding periods of hormonal fluctuation (e.g., adolescence), or implementing baseline screening and post-vaccination monitoring of thyroid function (e.g., TSH, anti-TPO antibodies), especially in individuals presenting with fatigue or unexplained weight changes, may warrant further evaluation to mitigate potential risks.

The underlying pathogenesis may involve adjuvant-mediated immune activation or molecular mimicry. Immunostimulatory adjuvants used in some vaccines have been implicated in triggering autoimmune phenomena (Perricone et al., 2016; Cohen Tervaert et al., 2023). Cervarix^®^ employs the AS04 adjuvant system. Studies demonstrate enhanced immunogenicity of AS04-adjuvanted vaccines compared to aluminum-only formulation (Garçon et al., 2011b). AS04 potentiates CD4^+^ T-cell proliferation and functional activation, thereby promoting memory B-cell differentiation (Garçon et al., 2011a). Notably, CD4^+^ T-cell hyperactivation constitutes a key pathogenic mechanism in Hashimoto’s thyroiditis development (Pyzik et al., 2015). Another hypothesis involves molecular mimicry and cross-reactivity between HPV antigens and thyroid autoantigens. Clinical observations have reported the presence of anti-thyroid peroxidase (TPO) antibodies in some vaccinated individuals (Colafrancesco et al., 2013), and experimental models have demonstrated that administration of thyroglobulin (Tg) or TPO can induce autoimmune thyroiditis in mice, suggesting these autoantigens play a critical role in HT pathogenesis (Pyzik et al., 2015).

Subgroup analysis stratified by HPV vaccine type revealed that 4vHPV exhibited the highest absolute number of thyroid-related adverse events reports (n = 187, accounting for 72.8% of total thyroid-related AEs), followed by 9vHPV (n = 54, 21.0%) and 2vHPV (n = 16, 6.2%). The distinct immunogenic and reactogenic profiles observed among the three HPV vaccines may arise from variations in their manufacturing platforms and adjuvant compositions (Leung et al., 2015). 2vHPV utilizes a baculovirus-mediated expression system in *Trichoplusia ni* Rix4446 cells to generate virus-like particles, which are combined with the AS04 adjuvant system (Garçon et al., 2011a). Mechanistic studies indicate that AS04 potentiates both humoral and cellular immunity through transient cytokine induction at the injection site (Giannini et al., 2006). This process enhances antigen-presenting cell activation and facilitates efficient antigen presentation to CD4^+^ T lymphocytes (Garçon et al., 2011a). In contrast,4vHPV and nonavalent 9vHPV vaccines are produced in the yeast *Saccharomyces cerevisiae*, coupled with amorphous aluminum hydroxyphosphate sulfate (AAHS) as an adjuvant (Garçon et al., 2007). AAHS exhibits superior binding affinity to L1 VLPs relative to conventional aluminum salts (Caulfield et al., 2007). However, there has not been any direct comparison between AS04 and AAHS adjuvants using identical HPV antigens. Autoimmune thyroiditis emerged as the predominant AE subtype across all vaccine groups, with potential pathogenic mechanisms possibly involving adjuvant-mediated immune activation or molecular mimicry, consistent with our overall study. Furthermore, hypothyroidism demonstrated a higher reporting proportion in the 4vHPV cohort (32.6%, 61/187), while no similar signal was observed in 2vHPV or 9vHPV subgroups. This potential association between hypothyroidism and 4vHPV vaccination may represent a novel finding. However, considering the study’s limitations and inherent complexity, we recommend conducting additional targeted investigations to validate and expand these observations, thereby enabling a comprehensive assessment of HPV vaccination’s impact on thyroid function.

Marked gender disparities were observed in this study. The vast majority of thyroid-related AEs occurred in females. Among male recipients, only autoimmune thyroid disorders exhibited stronger signals. Gender-specific vaccination rates may potentially confound the outcomes. This sex bias may reflect known differences in immune responses: females typically mount stronger humoral and cellular immunity, generate higher antibody titers to various antigens (especially T-cell independent), and display more rapid allograft rejection (Chiovato et al., 1993). Estrogen also modulates immune responses and is associated with increased susceptibility to anxiety, depression, and eating disorders—all of which have been linked to thyroid dysfunction and may contribute to increased AE reporting in women (Plaisier et al., 2008). Furthermore, AITD affects women up to four times more frequently than men, which may partially account for the observed imbalance (Hu et al., 2022). In summary, the immune and endocrine mechanistic differences discussed above provide an inherent biological explanation for the observed sex disparities, which constitutes the primary finding of this study. It is noteworthy that confounding factors such as vaccination rates and reporting behaviors may introduce additional potential bias, which could also exert certain influences on the results and still requires further investigation through more rigorously designed prospective studies in the future.

Furthermore, age-stratified analyses revealed heterogeneity in AE distribution, with thyroid-related events predominantly observed in younger vaccinees and no thyroid-related AEs were found in older vaccinees. This may be associated with age-related immunosenescence in older adults, a condition characterized by an impaired capacity to mount effective protective humoral and cellular immune responses against pathogens or vaccines (Crooke et al., 2019). People who can be vaccinated according to the guidelines are individuals aged 9–45 years (Zadeh Mehrizi et al., 2025). While high vaccination rates among the younger population may confound the results, our methodology employing signal intensity calculations effectively mitigates the impact of population size disparities. This phenomenon aligns with AITD epidemiology. AITD is most common between the ages of 30 and 60 (Wrońska et al., 2024). Additionally, depression and anxiety disorders are more likely to occur in middle age and childbearing years (Plaisier et al., 2008), while environmental factors such as stress play significant roles in AITD pathogenesis (Tomer and Huber, 2009; Ajjan and Weetman, 2015). The absence of significant thyroid AE signals in elderly subgroups remains incompletely elucidated pathophysiologically, warranting further investigation.

The time-to-onset analysis revealed a distinct bimodal distribution in thyroid-related adverse events post HPV vaccination. Early-phase risks were predominant, with 45.08% (95% CI: 39.2%–51.1%) of cases emerging within 30 days post-vaccination. A secondary peak comprising 16.06% (95% CI: 12.3%–20.5%) of cases manifested beyond 365 days. Our study identified that thyroid-related adverse events significantly associated with HPV vaccination were predominantly comprised of autoimmune diseases, including autoimmune thyroiditis (ROR = 4.26), autoimmune hypothyroidism (ROR = 11.65), and Hashimoto’s disease (ROR = 38.28), among others. This disease spectrum provides critical insights for interpreting the bimodal temporal distribution—particularly the second delayed peak. We hypothesize that the second peak may be primarily driven by biological mechanisms, whereby vaccination triggers a slowly progressive autoimmune process (such as Hashimoto’s thyroiditis) in genetically or immunologically susceptible individuals (Cohen Tervaert et al., 2023). Hashimoto’s thyroiditis typically exhibit an insidious onset, characterized by chronic and progressive inflammation and tissue damage (Ralli et al., 2020). The progression from immune initiation to the manifestation of typical clinical symptoms often takes months to years, which is highly consistent with the delayed peak observed in this study. On the other hand, this peak may also be partially attributable to reporting bias or diagnostic delay, wherein non-specific thyroid-related symptoms (e.g., fatigue, weight changes) are not promptly recognized and associated with vaccination, or are reported to pharmacovigilance systems long after the adverse event occurrence. The Weibull distribution analysis (shape parameter β = 0.54, 95% CI: 0.485-0.596; β < 1) indicates a monotonically decreasing hazard function over time, consistent with progressive risk reduction. These results suggested that we should be vigilant about the AEs associated with HPV vaccination in the first month, and early recognition of AEs caused by HPV vaccination is important, while also promptly addressing AEs that may arise. These findings need to be confirmed through prospective studies and long-term follow-up.

The strengths of our study are the ability of VAERS to accept reports from a variety of sources (healthcare providers, patients, and others) nationwide, in addition to rapid signal recognition and efficacy in monitoring rare adverse events.

## 5 Limitations

Notwithstanding, limitations exist. VAERS operates as a passive surveillance system collecting post-marketing adverse event reports from pharmaceutical manufacturers, physicians, regulatory agencies, and global vaccine recipients following FDA-approved administrations. Underreporting and delayed reporting persist despite mandatory AE reporting requirements. Submitted reports occasionally contain incomplete data and lack standardized reporting formats. Collectively, these constraints preclude robust causal inference. Nevertheless, VAERS remains invaluable for hypothesis generation, with subsequent validation recommended through more rigorous databases like the Vaccine Safety Datalink (VSD), which enables statistically stringent causality assessments.

## 6 Conclusion

This study identified disproportionality signals suggesting a potential association between HPV vaccination and various thyroid-related adverse events, particularly autoimmune thyroiditis and hypothyroidism. The majority of events were reported in females and younger individuals, with most occurring within the first 6 months post-vaccination. Subgroup and time-to-onset analyses further supported temporal clustering and demographic variability in AE reporting patterns. Although the underlying mechanisms remain to be fully elucidated, immune activation and molecular mimicry may contribute to the observed associations.

Given the limitations inherent in passive surveillance systems, these findings do not establish causality. However, they highlight the importance of continued pharmacovigilance and suggest that targeted monitoring of thyroid function in susceptible individuals may be warranted. Further prospective studies and mechanistic investigations are needed to clarify the potential immunological links between HPV vaccination and thyroid autoimmunity.

## Data Availability

All data analyzed in this study were obtained from the publicly available Vaccine Adverse Event Reporting System (VAERS; https://vaers.hhs.gov/). Derived data and analysis code are available from the corresponding author on reasonable request.
