# Supplementary material for: HPV vaccine-related thyroid adverse events: temporal patterns and reporting trends
Source: Front Pharmacol. 2025 Nov 11;16:1664697. doi: 10.3389/fphar.2025.1664697 (PMC12644041; doi:10.3389/fphar.2025.1664697)
Supplement: Supplementary file 1 [file Supplementaryfile1.docx]

Supplementary Material

# Supplementary Tables

## Supplementary Table 1

**Table S1 Multiple pharmacovigilance signal detection algorithms.**

| **Name of Algorithm** | **Formula** | **Positive Signal Criteria** |
| --- | --- | --- |
| **ROR** | ROR=ad/bc | Lower Limit of 95%CI>1, N≥3 |
|  | 95%CI=e ^ln(ROR)±1.96(1/a+1/b+1/c+1/d)^0.5^ |  |
| **PRR** | PRR=(a(c+d))/(c(a+b)) | PRR≥2, X²≥4, N≥3 |
|  | X²=[(ad-bc)^2](a+b+c+d)/[(a+b)(c+d)(a+c)(b+d)] |  |
| **BCPNN** | IC=log_2_a(a+b+c+d)(a+c)(a+b) | IC_025_>0 |
|  | IC_025_=e ^ln(IC)-1.96(1/a+1/b+1/c+1/d)^0.5^ |  |
| **MGPS** | EBGM=a(a+b+c+d)/((a+c)/(a+b)) | EBGM05>2, N>0 |
|  | EBGM05=e ^ln(EBGM)-1.64(1/a+1/b+1/c+1/d)^0.5^ |  |

*Abbreviations:* a, number of reports containing both the suspect drug and the suspect adverse drug reaction; b, number of reports containing the suspect adverse drug reaction with other medications (except the drug of interest); c, number of reports containing the suspect drug with other adverse drug reactions (except the event of interest); d, number of reports containing other medications and other adverse drug reactions. ROR, reporting odds ratio; CI, confidence interval; N, the number of co-occurrences; PRR, proportional reporting ratio; χ2, chi-squared; BCPNN, Bayesian confidence propagation neural network; IC, information component; IC025, the lower limit of the 95% CI of the IC; E (IC), the IC expectations; V (IC), the variance of IC; MGPS, multi-item gamma Poisson shrinker; EBGM, empirical Bayesian geometric mean; EBGM05, the lower limit of the 95% CI of EBGM.

## Supplementary Table 2

Table S2 Signal detection of HPV vaccine-associated thyroid AE.

| **Preferred terms** | **N** | **ROR (95% two-­sided CI)** | **PRR (95% two-­sided CI)** | **χ2** | **IC(IC025)** | **EBGM (EBGM05)** |
| --- | --- | --- | --- | --- | --- | --- |
| HASHITOXICOSIS | 1 | 38.28 ( 2.39 - 612.06 ) | 38.28 (35.51 - 41.05) | 18.15 | 4.3 ( 1.66 ) | 19.64 ( 1.93 ) |
| HYPOTHYROIDIC GOITRE | 1 | 38.28 ( 2.39 - 612.06 ) | 38.28(35.51 - 41.05) | 18.15 | 4.3 ( 1.66 ) | 19.64 ( 1.93 ) |
| THYROIDITIS CHRONIC | 1 | 12.76 ( 1.33 - 122.68 ) | 12.76(10.5 - 15.02) | 8.13 | 3.3 ( 0.88 ) | 9.82 ( 1.48 ) |
| AUTOIMMUNE HYPOTHYROIDISM | 7 | 11.65 ( 5 - 27.15 ) | 11.65(10.8 - 12.5) | 52.25 | 3.2 ( 2.05 ) | 9.17 ( 4.52 ) |
| THYROID CANCER | 10 | 7.22 ( 3.67 - 14.2 ) | 7.22 (6.55 - 7.9) | 45.1 | 2.64 ( 1.7 ) | 6.24 ( 3.54 ) |
| EUTHYROID SICK SYNDROME | 1 | 6.38 ( 0.77 - 53 ) | 6.38(4.26 - 8.5) | 3.89 | 2.49 ( 0.21 ) | 5.61 ( 0.95 ) |
| THYROID CYST | 8 | 5.89 ( 2.8 - 12.4 ) | 5.89(5.15 - 6.63) | 28.14 | 2.39 ( 1.36 ) | 5.24 ( 2.81 ) |
| AUTOIMMUNE THYROIDITIS | 103 | 4.26 ( 3.48 - 5.23 ) | 4.26 (4.06 - 4.47) | 231.47 | 1.98 ( 1.68 ) | 3.94 ( 3.32 ) |
| PAPILLARY THYROID CANCER | 3 | 4.25 ( 1.29 - 14.02 ) | 4.25(3.06 - 5.45) | 6.72 | 1.97 ( 0.44 ) | 3.93 ( 1.45 ) |
| THYROID DISORDER | 41 | 2.76 ( 2.01 - 3.79 ) | 2.76 (2.45 - 3.08) | 43.03 | 1.4 ( 0.94 ) | 2.64 ( 2.03 ) |
| HYPOTHYROIDISM | 82 | 2.46 ( 1.97 - 3.08 ) | 2.46 (2.24 - 2.68) | 66.78 | 1.25 ( 0.92 ) | 2.37 ( 1.97 ) |
| GOITRE | 22 | 1.93 ( 1.26 - 2.96 ) | 1.93 (1.5 - 2.36) | 9.41 | 0.92 ( 0.3 ) | 1.89 ( 1.32 ) |
| BASEDOW'S DISEASE | 33 | 1.47 ( 1.04 - 2.08 ) | 1.47 (1.12 - 1.81) | 4.73 | 0.54 ( 0.03 ) | 1.45 ( 1.08 ) |

*Abbreviations:* CI, confidence interval; EBGM, empirical Bayesian geometric mean; IC, information component; IC025 and EBGM05, lower one‐sided for IC and EBGM, respectively; N, number of adverse event reported; PRR, proportional reporting ratio; ROR, reporting odds ratio; χ 2, chi‐squared.

^a^ Adhering to the four algorithms.

## Supplementary Table 3

**Table S3 Detection of thyroid adverse event signals associated with HPV vaccination stratified by HPV vaccine type**

| **Subgroup** | **Preferred terms** | **N** | **χ2** | **ROR (95%two-**­sided CI) | | **PRR (95%two-**­sided CI) | **EBGM (EBGM05)** | | | **IC (IC025)** | |  |
| --- | --- | --- | --- | --- | --- | --- | --- | --- | --- | --- | --- | --- |
| **Bivalent HPV Vaccine.** | AUTOIMMUNE THYROIDITIS | 10 | 14.48 | 3.14 ( 1.69 - 5.86 ) | | 3.14 ( 14.48 ) | 3.12 ( 1.85 ) | | 1.64 ( 0.77 ) | | |  |
|  | THYROID CANCER | 1 | 3.3 | 5.16 ( 0.72 - 37.23 ) | | 5.16 ( 3.3 ) | 5.1 ( 0.98 ) | | 2.35 ( 0.28 ) | | |  |
|  | HASHITOXICOSIS | 1 | 159.01 | 320.03 ( 20.02 - 5116.91 ) | | 320.02 ( 159.01 ) | 160.51 ( 15.78 ) | | 7.33 ( 4.69 ) | | |  |
|  | THYROID CYST | 1 | 3.55 | 5.42 ( 0.75 - 39.15 ) | | 5.42 ( 3.55 ) | 5.35 ( 1.02 ) | | 2.42 ( 0.35 ) | | |  |
|  | THYROIDITIS CHRONIC | 1 | 78.51 | 106.68 ( 11.1 - 1025.62 ) | | 106.67 ( 78.51 ) | 80.26 ( 12.08 ) | | 6.33 ( 3.91 ) | | |  |
|  | AUTOIMMUNE HYPOTHYROIDISM | 1 | 8.82 | 11.04 ( 1.5 - 81.02 ) | | 11.04 ( 8.82 ) | 10.7 ( 2.02 ) | | 3.42 ( 1.31 ) | | |  |
| **Quadrivalent HPV Vaccine** | AUTOIMMUNE THYROIDITIS | 67 | 156.75 | 4.27 ( 3.33 - 5.47 ) | | 4.27 ( 156.75 ) | 4.06 ( 3.3 ) | | 2.02 ( 1.66 ) | | |  |
|  | HYPOTHYROIDISM | 61 | 71.46 | | 2.88 ( 2.23 - 3.72 ) | 2.88 ( 71.46 ) | | 2.79 ( 2.25 ) | 1.48 ( 1.11 ) | |  |  |
|  | THYROID DISORDER | 27 | 30.76 | | 2.84 ( 1.93 - 4.18 ) | 2.84 ( 30.76 ) | | 2.76 ( 2 ) | 1.46 ( 0.91 ) | |  |  |
|  | GOITRE | 14 | 6.09 | | 1.93 ( 1.13 - 3.29 ) | 1.93 ( 6.09 ) | | 1.9 ( 1.22 ) | 0.93 ( 0.17 ) | |  |  |
|  | THYROID CANCER | 6 | 24.97 | | 6.44 ( 2.78 - 14.95 ) | 6.44 ( 24.97 ) | | 5.93 ( 2.93 ) | 2.57 ( 1.42 ) | |  |  |
|  | AUTOIMMUNE HYPOTHYROIDISM | 5 | 43.02 | | 12.24 ( 4.69 - 31.98 ) | 12.24 ( 43.02 ) | | 10.37 ( 4.64 ) | 3.37 ( 2.09 ) | |  |  |
|  | THYROID NEOPLASM | 2 | 3.38 | | 3.5 ( 0.84 - 14.54 ) | 3.5 ( 3.38 ) | | 3.36 ( 1.02 ) | 1.75 ( 0.02 ) | |  |  |
|  | PAPILLARY THYROID CANCER | 2 | 4.86 | | 4.37 ( 1.04 - 18.36 ) | 4.37 ( 4.86 ) | | 4.15 ( 1.25 ) | 2.05 ( 0.31 ) | |  |  |
|  | EUTHYROID SICK SYNDROME | 1 | 7.12 | | 10.2 ( 1.23 - 84.75 ) | 10.2 ( 7.12 ) | | 8.89 ( 1.51 ) | 3.15 ( 0.87 ) | |  |  |
|  | HYPOTHYROIDIC GOITRE | 1 | 29.62 | | 61.22 ( 3.83 - 978.78 ) | 61.22 ( 29.62 ) | | 31.11 ( 3.06 ) | 4.96 ( 2.33 ) | |  |  |
|  | PARATHYROID GLAND ENLARGEMENT | 1 | 8.6 | | 12.24 ( 1.43 - 104.8 ) | 12.24 ( 8.6 ) | | 10.37 ( 1.72 ) | 3.37 ( 1.06 ) | |  |  |
| **The 9-Valent HPV Vaccine** | AUTOIMMUNE THYROIDITIS | 22 | 52.56 | | 4.2 ( 2.76 - 6.42 ) | 4.2 ( 52.56 ) | | 4.13 ( 2.9 ) | 2.05 ( 1.44 ) | |  |  |
|  | THYROID MASS | 9 | 33.18 | | 5.62 ( 2.89 - 10.9 ) | 5.62 ( 33.18 ) | | 5.49 ( 3.15 ) | 2.46 ( 1.53 ) | | | |
|  | THYROID DISORDER | 8 | 7.49 | | 2.56 ( 1.27 - 5.14 ) | 2.56 ( 7.49 ) | | 2.54 ( 1.42 ) | 1.34 ( 0.37 ) | | | |
|  | GOITRE | 7 | 9.09 | | 2.98 ( 1.41 - 6.29 ) | 2.98 ( 9.09 ) | | 2.95 ( 1.58 ) | 1.56 ( 0.53 ) | | | |
|  | THYROID CYST | 5 | 71.18 | | 17.47 ( 6.99 - 43.65 ) | 17.47 ( 71.18 ) | | 16.1 ( 7.48 ) | 4.01 ( 2.77 ) | | | |
|  | THYROID CANCER | 3 | 22.04 | | 9.61 ( 3.01 - 30.64 ) | 9.61 ( 22.04 ) | | 9.2 ( 3.49 ) | 3.2 ( 1.71 ) | | | |
|  |  |  |  | |  |  | |  |  | | | |

*Abbreviations:* CI, confidence interval; EBGM, empirical Bayesian geometric mean; IC, information component; IC025 and EBGM05, lower one‐sided for IC and EBGM, respectively; N, number of adverse event reported; PRR, proportional reporting ratio; ROR, reporting odds ratio; χ 2, chi‐squared.

^a^ Adhering to the four algorithms
